# Quantification of extrusion-induced excavation volume in shield tunnelling through squeezing cohesive-frictional ground

**DOI:** 10.1038/s41598-025-92930-9

**Published:** 2025-03-11

**Authors:** Pferdekämper Thomas, Anagnostou Georgios

**Affiliations:** ETH Zurich, Zurich, Switzerland

**Keywords:** Excavation volume, Squeezing ground, TBM advance, Face extrusion, Cohesive-frictional ground, TBM-ground interaction, Solid Earth sciences, Civil engineering

## Abstract

When tunnelling through weak and highly deformable ground, relevant deformations occur ahead of the tunnel face and result in face displacements that are opposite to the advance direction (“extrusion”). In mechanised tunnelling, the cutterhead continuously removes the ground extruding at the tunnel face during advance. Therefore, the total excavation volume is higher than the volume that corresponds to the tunnel cross-section area. Previous investigations by the authors showed that the additional, extrusion-induced excavation volume may be considerable in the case of purely cohesive ground. This paper extends this work for the case of cohesive-frictional ground. Firstly, we quantify the extrusion-induced excavation volume for a wide parameter range by means of a numerical model that uses re-meshing to consider the continuous re-profiling taking place at the tunnel face during advance. The results of the performed parametric study are summarised in a single dimensionless design chart. Secondly, we derive a simple closed-form expression, which, despite the underlying simplifications, allows for an accurate and rapid quantification of the extrusion-induced excavation volume. The results show that face extrusion has an effect on excavation volume in the cohesive-frictional case, although less pronounced than when tunnelling through low-permeability clays with purely cohesive short-term strength. The study suggests that if the additional excavation volume due to face extrusion is not taken into account in the design of the mechanised advance, it may (a) pose logistical and disposal challenges and (b) lead to erroneous conclusions about the stability of the tunnel face, both of which affect the rate of tunnel advance.

## Introduction

**Fig. 1:**
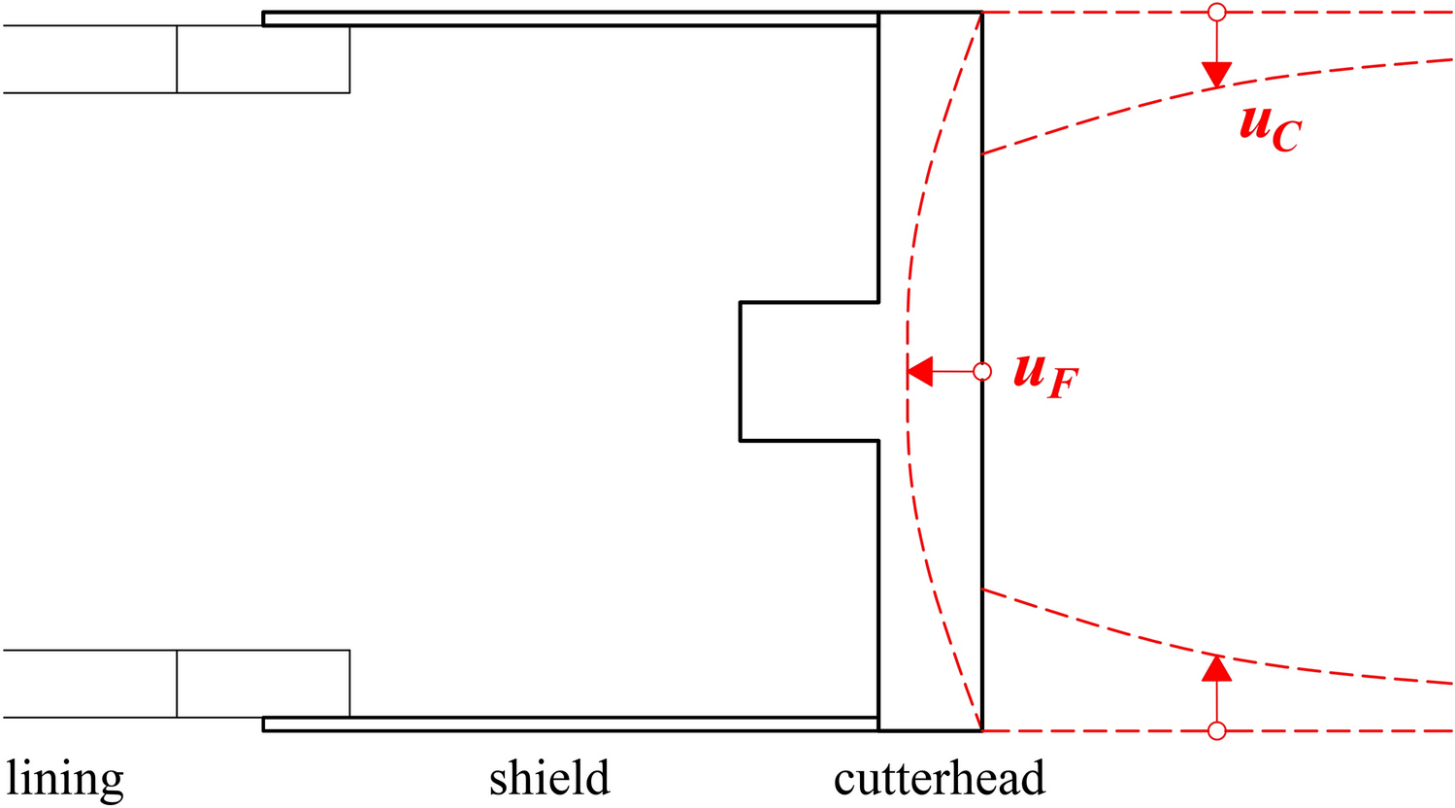
Pre-convergence *u*_*C*_ and face extrusion *u*_*F*_.

Squeezing manifests itself by a relevant reduction of the tunnel cross-section (“convergence”) and an axial displacement of the tunnel face (“extrusion”) and can occur when tunnelling through highly deformable, low-strength rocks such as kakirite, phyllite, schist, serpentine, mudstone, tuff, flysch and chemically altered igneous rocks^1^. In shield tunnelling, the deformations can only be assessed indirectly as neither the shield extrados nor the tunnel face are accessible during the advance process. Nevertheless, rapidly developing convergences in the shield area can be inferred from an increase in the thrust force that is necessary to overcome shield skin friction during advance of the tunnel boring machine (TBM), while a large extrusion of the tunnel face could be inferred from an increase in the torque demand or in the weight of the spoil material. The latter is because the machine excavates (continuously during advance and additionally to the volume that corresponds to the tunnel cross-section area) the ground entering the cross-section ahead of the face (pre-convergence; *u*_*C*_ in Fig. 1) and extruding at the face (*u*_*F*_ in Fig. 1).

Rapid technical progress in terms of TBM thrust, torque and support pressure^2^ has made it possible to use TBMs in increasingly difficult conditions. Under heavy squeezing conditions, the interaction between the cutterhead and the extruding tunnel face can be crucial for the feasibility of TBM drives, in addition to the potential hazard of shield jamming^3^.

Since no previous work in the literature has addressed the question of the excavation volume during tunnel advance under heavily squeezing conditions, the authors have developed a computational method that specifically considers the excavation of the material that continuously extrudes at the face during TBM tunnelling^3–6^. Unlike typical numerical methods used to study the excavation advance and support sequence, which do not take into account the ground entering the cavity at the face, the developed method uses re-meshing and stress re-mapping techniques to account for the re-profiling of the tunnel face due to the cutterhead rotation. Using this method, the case of purely cohesive material (a model for the behaviour of low-permeability clayey ground during TBM advance) was analysed and it was shown that the additional excavation volume caused by extrusion can be considerable. An excessive excavation volume would affect mucking out, TBM performance and torque requirements, and in extreme cases it would even lead to jamming of the cutterhead (see^7^ for the experience of the Gilgel Gibe II tunnel, a particularly demanding project in this respect). Furthermore, a large over-excavation can falsely indicate an unstable tunnel face, leading to faulty decision-making during TBM operations^8^.

This paper extends the previous works to consider cohesive-frictional grounds obeying the Mohr–Coulomb yield criterion. The extrusion-induced excavation volume is quantified numerically and analytically. Using the aforementioned computational model, which is outlined in Section “Computational model”, a comprehensive parametric study covering the entire practically relevant parameter range is performed and, based upon its results and some fundamental properties of the numerical solutions, a dimensionless design chart is worked-out (Sections “Parametric study” and “Numerical results”). Furthermore, a closed-form solution for the additional extrusion-induced excavation volume is derived, the predictions of which—despite the simplicity of the proposed equation and the complexity of the problem under consideration—agree very well with the numerical results (Section “Analytical solution”), thus allowing for a rapid and accurate performance of parametric studies for given geotechnical conditions, as illustrated by an application example in Section “Application example”. Finally, the case of cohesive-frictional grounds is compared with that of a purely cohesive material, showing that cohesive-frictional materials are less prone to severe over-excavation than purely cohesive ones (Section “Comparison with purely cohesive ground”).

## Computational model

**Fig. 2:**
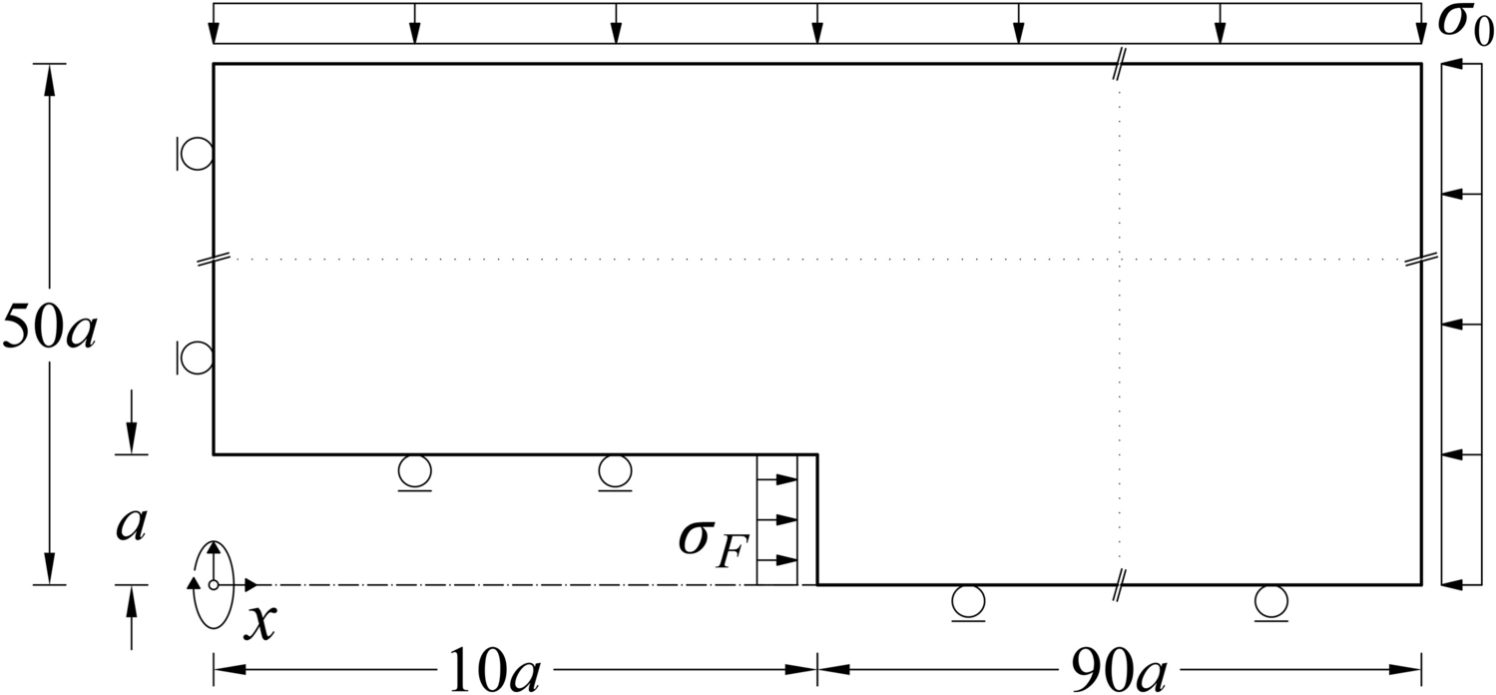
Model layout.

We consider the axially symmetric problem of a deep cylindrical tunnel of radius *a* in a homogeneous and isotropic stress field (in-situ stress *σ*_0_). The ground is taken as linearly elastic (Young’s modulus *E* and Poisson’s ratio *ν*) and perfectly plastic obeying the Mohr–Coulomb yield criterion (friction angle *φ* and uniaxial compressive strength *f*_*c*_). The flow rule is non-associated and defined by the dilation angle *ψ*. Figure 2 shows the computational domain. The advance of the TBM is simulated starting from the left boundary for a length of 10*a* to mitigate boundary effects and reach a steady state with respect to the advancing face. The case of closed-shield tunnelling is also considered, assuming that a support pressure acts upon the tunnel face (*σ*_*F*_ > 0). On account of the high deformability of the ground, we assume for simplicity that the lining and shield are rigid, which allows modelling them by fixing the nodes after every excavation step in the radial direction. Previous numerical analyses^5^ investigated the influence of the TBM overcut on the over-excavation by considering a radial shield gap and showed that the excavation volume increases slightly with increasing TBM overcut.

The computations are performed with the commercial finite element software Abaqus®^9^. TBM advance is simulated by means of discrete excavation steps of length *Δx*, whereby the results for continuous advance (excavation step length *Δx* → 0) are obtained by linear extrapolation from the numerical results for *Δx* = *a*/2, *a*/4, *a*/8^4^.

In addition to the volume of the core *V*_0_, the excavation of the extruding material *ΔV* during TBM advance is taken into account by means of successive re-profiling rounds within every excavation step, with each re-profiling round involving model re-meshing and stress re-mapping^5^. A schematic representation of the numerical re-profiling algorithm within a step-by-step computation can be found in^6^.

## Parametric study

The displacements *u* depend on all problem parameters, i.e. the five material constants, the initial stress *σ*_*0*_, the face support pressure *σ*_*F*_ and the tunnel radius *a*:

1 $$u = f\left( {E,\;\nu ,\;\varphi ,\;f_{c} ,\;\psi ,\;\sigma_{0} ,\;\sigma_{F} ,\;a} \right),$$

but, as shown by^10^ and confirmed by other works^11,12^, the number of significant parameters can be reduced by three by, (i), performing a dimensional analysis; (ii), using Caquot’s transformation of normal stresses^13^, i.e.

2 $$\overline{\sigma } = \sigma + f_{c} /(m - 1),$$

where the constant *m* = (1 + sin*φ*)/(1 − sin*φ*); and, (iii), considering that the displacements *u* are inversely proportional to the Young’s modulus *E*. The latter is strictly true only for small strains, but will be confirmed later based upon the numerical results. Then, all displacements can be expressed in the following dimensionless form:

3 $$\frac{u}{a}\frac{E}{{\overline{\sigma }_{0} }} = f\left( {\frac{{\overline{\sigma }_{F} }}{{\overline{\sigma }_{0} }},\;\varphi ,\;\psi ,\;\nu } \right).$$

Table 1 shows the considered parameter ranges. The corresponding $\overline{\sigma }_{F} /\overline{\sigma }_{0}$- values cover a wide range of conditions, from elastic (non-squeezing) to heavily squeezing. Young’s modulus *E*, strength *f*_*c*_, in-situ stress *σ*_*0*_ and face pressure *σ*_*F*_ have been varied independently from each other in some calculations to verify that *u* is inversely proportional to *E*, and that *σ*_*0*_, *σ*_*F*_ and *f*_*c*_ are not independent significant parameters.

**Table 1:** Values considered in the parametric study.

In-situ stress	*σ*_0_ = 5–20 MPa
Face support pressure	*σ*_*F*_ = 0–2 MPa
Young’s modulus	*E* = 0.15–2 GPa
Poisson’s ratio	*ν* = 0.3
Friction angle	*φ* = 15°–35°
Dilation angle^a^	*ψ* = max(1°; *φ*—20°)
Uniaxial compressive strength^b^	*f*_*c*_ = 0.05–0.5 *σ*_0_

^a^After^15^; cf.^16^.

^b^The corresponding normalised values ${{\overline{\sigma }_{F} } \mathord{\left/ {\vphantom {{\overline{\sigma }_{F} } {\overline{\sigma }_{0} }}} \right. \kern-0pt} {\overline{\sigma }_{0} }}$ = 0.018–0.5

In particular, the Young’s modulus *E* ranges from highly deformable to stiff rocks (*E* = 0.15–2 GPa). The in-situ stress *σ*_0_ covers an overburden approximately between 200 and 1000 m (for a unit weight *γ* ≈ 20–25 kN/m^3^) and the face support pressure *σ*_*F*_ ranges from zero (open mode operation) and 2 MPa, with the current maximum design and applied face support pressures being 1.7 MPa and 1.4 MPa, respectively^14^.

The wide range of parameters adopted provides a practical answer to the well-known variability of the squeezing deformations associated with ground heterogeneity and anisotropy at different scales (bedding, faults, and schistosity) and non-isotropic stress conditions. In addition, the typical response of the ground in squeezing conditions is delayed due to ground rheology (and sometimes also due to consolidation). The assumption of instantaneous deformations is therefore appropriate in the preliminary design stage, as the rheological behaviour of the ground is favourable with respect to the excavation volume during tunnel advance.

## Numerical results

**Fig. 3:**
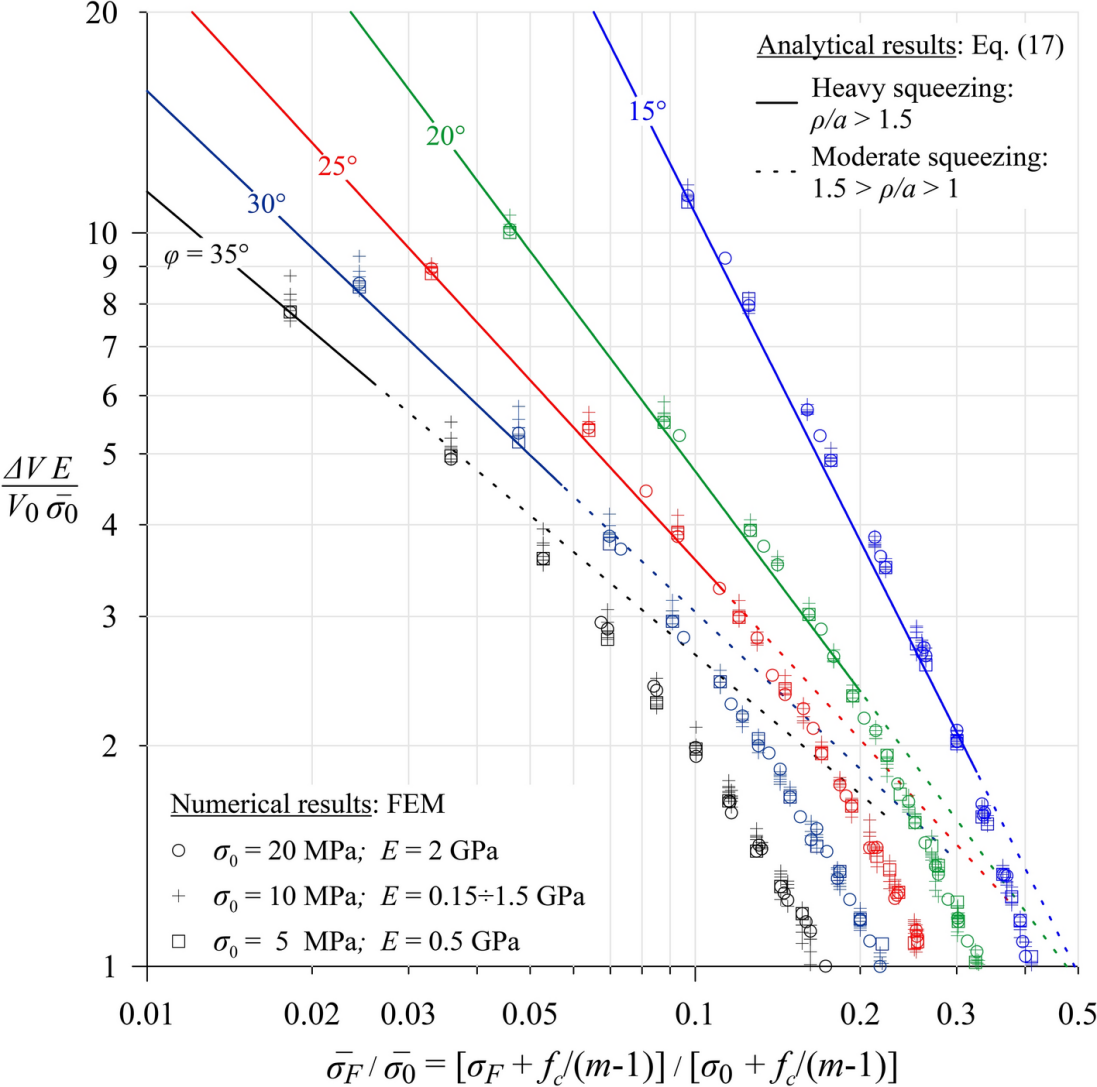
Normalized additional extrusion-induced excavation volume over normalised face support pressure.

As the values of the two last parameters of Eq. (3) have been fixed and the extrusion-induced excavation volume is obtained by integrating the displacements over the excavation boundary, the results of the parametric study can be expressed as

4 $$\frac{\Delta V}{{V_{0} }}\frac{E}{{\overline{\sigma }_{0} }} = f\left( {\frac{{\overline{\sigma }_{F} }}{{\overline{\sigma }_{0} }},\varphi } \right),$$

which is presented in Fig. 3 using marked points. (The continuous lines have been obtained analytically and will be discussed later.)

The numerical results confirm the validity of considering $\overline{\sigma }_{F} /\overline{\sigma }_{0}$ and $uE/a/\overline{\sigma }_{0}$ as significant parameters, as they only exhibit a small scatter for different Young’s moduli.

Figure 3 clearly illustrates the very favourable effect of a high friction angle. The linear trend of the results in the upper part of the double-logarithmic diagram points to a power law, which are a reminder of the simplified elastoplastic ground response curve after^17^ and provides a motivation to analytically study the problem.

## Analytical solution

**Fig. 4:**
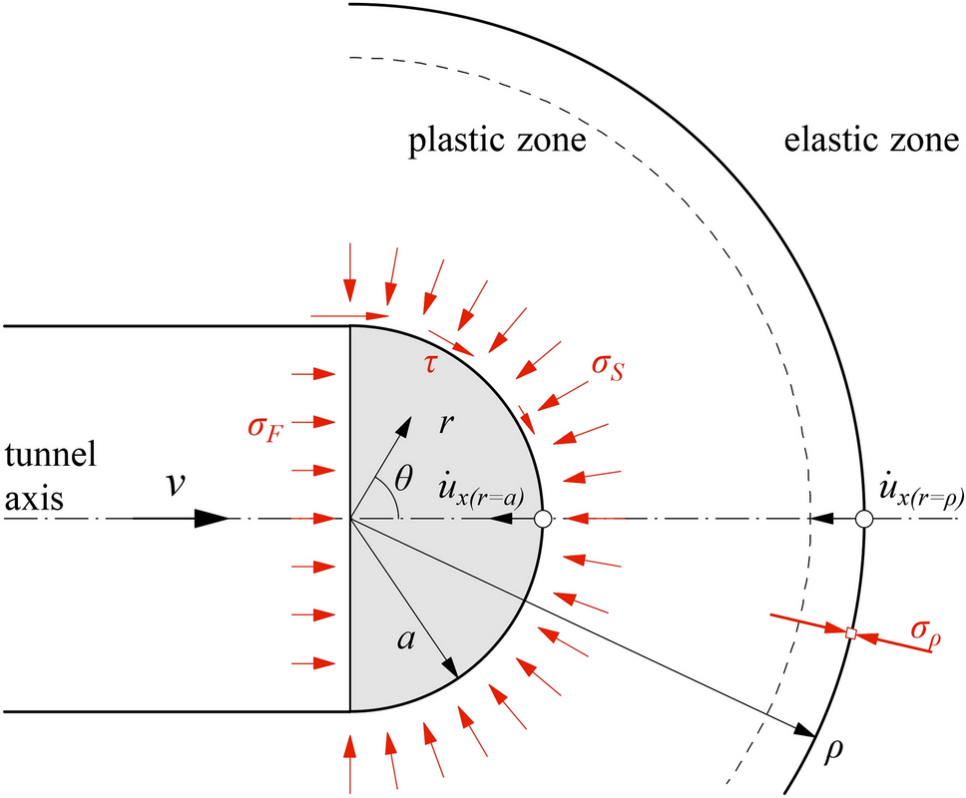
Considered system consisting of half of a sphere and half of a thick-walled hollow sphere ahead of the tunnel face.

**Fig. 5:**
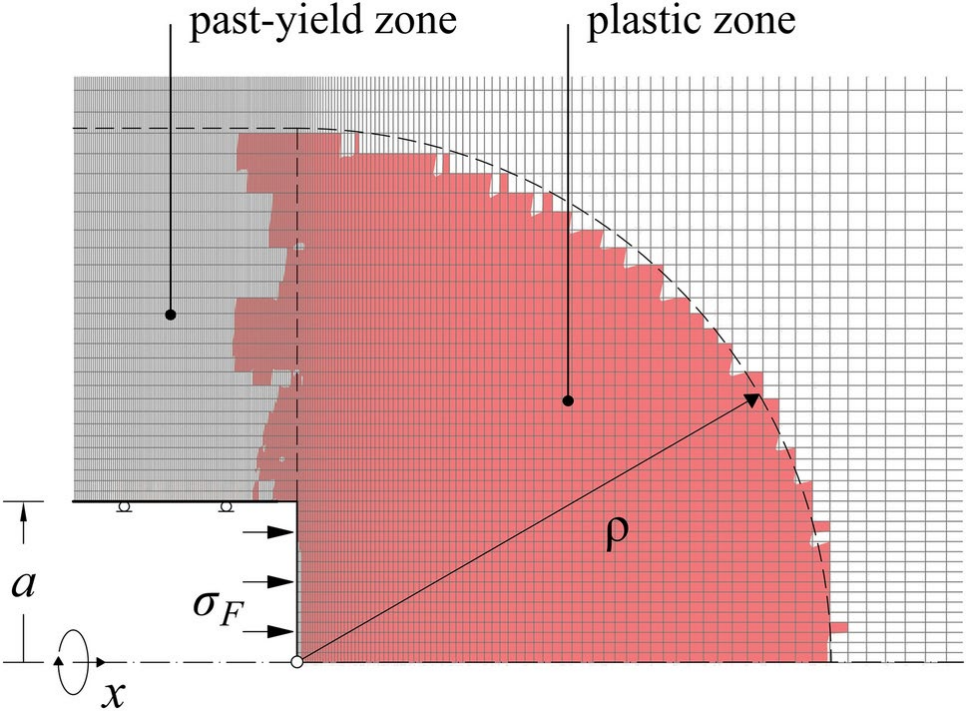
Numerically obtained plastic zone (*σ*_0_ = 20 MPa; *σ*_*F*_ = 0 MPa; *E* = 2 GPa; *ν* = 0.3; *φ* = 15°; *f*_*c*_ = 1.5 MPa; *ψ* = 1°; *Δx* = *a*/8).

Figure 4 shows the geometry and boundary conditions of the examined problem. As suggested in the literature^18^ and in previous investigations by the authors for a purely cohesive ground^5^ and justified by numerical results (see numerical example of Fig. 5), the analytical solution is derived by approximating the plastic zone in front of the advancing tunnel face by a half sphere.

The considered half-spherical plastic zone (Fig. 4) consists of an inner region (half of a sphere of radius *a*) and an outer region (half of a thick-walled hollow sphere with inner radius *a* and outer radius *ρ*).

### Static considerations

Firstly, the outer region will be considered. Assuming spherical symmetry of stress and strain, we obtain from the equilibrium and the Mohr–Coulomb yield condition the radius *ρ* of the plastic zone^11^:

5 $$\frac{\rho }{a} = \left( {\frac{{\overline{\sigma }_{\rho } }}{{\overline{\sigma }_{s} }}} \right)^{{\frac{1}{{2\left( {m - 1} \right)}}}} ,$$

where *σ*_*s*_ is the support pressure exerted by the inner region upon the outer region; and *σ*_*ρ*_ denotes the radial stress at the elastoplastic interface. The latter is determined considering that the stress state at *r* = *ρ* fulfils both Kirsch’s solution and the Mohr–Coulomb yield condition, and reads as follows^11^:

6 $$\overline{\sigma }_{\rho } = \frac{3}{2m + 1} \cdot \overline{\sigma }_{0} .$$

The support pressure *σ*_*s*_ is determined from the axial equilibrium of the inner region (see Fig. 4):

7 $$\overline{\sigma }_{s} = \overline{\sigma }_{F} + \int\limits_{0}^{\pi /2} {\tau \left( \theta \right) \cdot \sin^{2} \theta } \cdot d\theta ,$$

where *τ* denotes the shear stress. The simplest possible assumption concerning the shear stress *τ* is that it increases linearly with the angle *θ* from zero (at the tunnel axis) to its maximum value *τ*_*π/2*_ at the edge of the face:

8 $$\tau \left( \theta \right) = \tau_{\pi /2} \frac{2\theta }{\pi }.$$

**Fig. 6:**
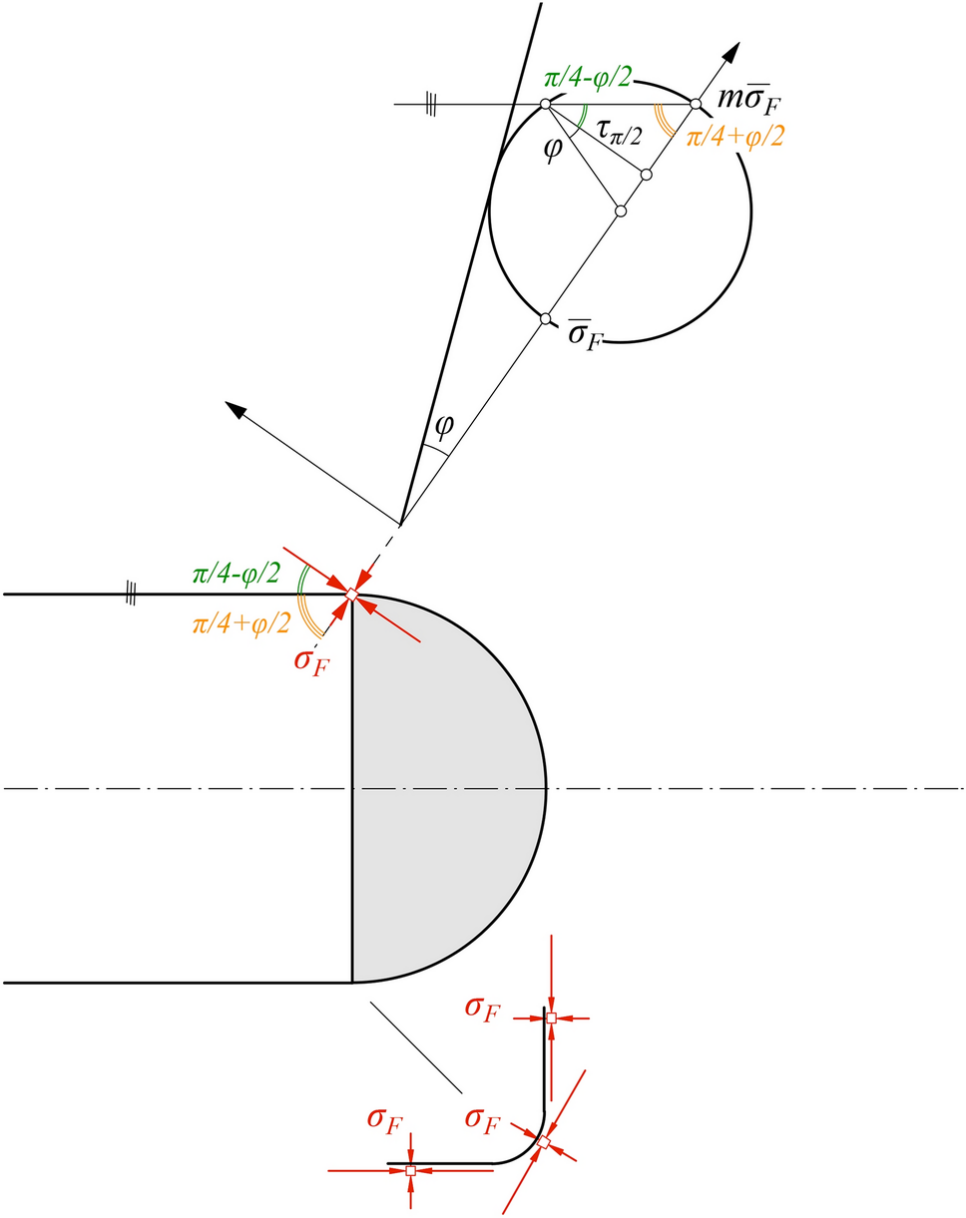
Assumption about the stress state at the edge of the face and determination of the shear stress.

The shear stress *τ*_*π/2*_ is determined by making the following two simplifying assumptions about the stress state at the edge of the face: (i) the maximum principal stress forms an angle of *π*/4–*φ*/2 to the direction of shearing, which on account of the movement of the hemisphere towards the cavity coincides with the tunnel axis (Fig. 6); (ii) the minimum principal stress is equal to the face support pressure *σ*_*F*_.

The first simplifying assumption is reasonable because the dilation angle of squeezing rocks is usually very small^19^. The second assumption is also reasonable because the tunnel face and wall are shear stress-free and, consequently, the principal directions are oriented tangentially and perpendicularly to the cavity boundary which is supported by *σ*_*F*_ (see sketch at the bottom of Fig. 6), regardless of the rotation of the principal axes at the corner. These two assumptions completely define the stress state and allow determining the shear stress (e.g., graphically; see Mohr diagram at the top of Fig. 6):

9 $$\tau_{\pi /2} = \frac{m - 1}{2} \cdot \overline{\sigma }_{F} \cdot \cos \varphi .$$

From Eqs. (7)–(9) we obtain:

10 $$\frac{{\bar{\sigma }_{s} }}{{\bar{\sigma }_{F} }} = \mu = 1 + \left( {\frac{\pi }{8} + \frac{1}{{2\pi }}} \right) \cdot \frac{{\sin \varphi \cos \varphi }}{{1 - \sin \varphi }}\left( { = 1.19 - 1.61\;{\text{for}}\;\varphi = 15^{\circ } - 35^{\circ } } \right)$$

Introducing *σ*_*ρ*_ and *σ*_*s*_ from Eqs. (6) and (10) in Eq. (5) provides the radius of the plastic zone as a function of the transformed in-situ stress, the transformed face support pressure and the friction angle:

11 $$\frac{\rho }{a} = \left( {\frac{3}{{\mu \left( {2m + 1} \right)}}\frac{{\overline{\sigma }_{0} }}{{\overline{\sigma }_{F} }}} \right)^{{\frac{1}{{2\left( {m - 1} \right)}}}} .$$

### Kinematic considerations

The outer region of Fig. 4 moves together with the advancing tunnel heading with the rate *v*. The rate of ground volume $\dot{V}_{\rho }$ passing through the hemispherical elastoplastic interface is equal to $2\pi \rho^{2} \dot{u}_{x(r = \rho )}$, where $\dot{u}_{x(r = \rho )}$ is the displacement rate at the elastoplastic interface. Considering steady conditions with respect to the advancing heading, $\dot{u}_{x(r = \rho )} = - v{{\partial u_{x} } \mathord{\left/ {\vphantom {{\partial u_{x} } {\partial x_{(r = \rho )} }}} \right. \kern-0pt} {\partial x_{(r = \rho )} }}$^20^ and, consequently,

12 $$\dot{V}_{\rho } = 2\pi \rho^{2} \cdot v \cdot \varepsilon_{x(r = \rho )} ,$$

where the last r.h.s. term denotes the axial strain at the elastoplastic boundary and reads as follows^21^:

13 $$\varepsilon_{x(r = \rho )} = \frac{1 + \nu }{E} \cdot \left( {\overline{\sigma }_{0} - \overline{\sigma }_{\rho } } \right).$$

The rate of the ground volume $\Delta \dot{V}$ that extrudes at the tunnel face is practically equal to the rate of the ground volume $\dot{V}_{a}$ that passes through the inner boundary of the outer region, but the rate $\dot{V}_{a}$ is in general greater than $\dot{V}_{\rho }$, due to the plastic dilatancy experienced by the material.

Under the simplifying assumption of negligible elastic strains in the plastic zone^17^, the radial and tangential strains fulfil the flow rule

14 $$d\varepsilon_{r} + 2\kappa \cdot d\varepsilon_{t} = 0,$$

where the so-called “loosening factor” *κ* = (1 + sin*ψ*)/(1 − sin*ψ*). In combination with the kinematic relations, Eq. (14) in results in a differential equation for the radial displacement field, the solution of which provides the following simple equation for the ratio of the displacement rates at the inner and outer boundary of the plastic zone:

15 $$\frac{{\dot{u}_{x(r = a)} }}{{\dot{u}_{x(r = \rho )} }} = \left( {\frac{\rho }{a}} \right)^{2\kappa } .$$

The ratio of the volumes entering and exiting the body during advance then reads as follows:

16 $$\frac{{\dot{V}_{a} }}{{\dot{V}_{\rho } }} = \frac{{2\pi a^{2} \cdot \dot{u}_{x(r = a)} }}{{2\pi \rho^{2} \cdot \dot{u}_{x(r = \rho )} }} = \left( {\frac{\rho }{a}} \right)^{2(\kappa - 1)} ,$$

which in combination with Eqs. (10)–(13) leads to the following expression for the additional, extrusion-induced excavation volume, normalised by the theoretical tunnel volume *V*_*0*_ (= π*a*^2^ per linear meter):

17 $$\frac{\Delta V}{{V_{0} }} = \frac{{4\left( {1 + \nu } \right)\left( {m - 1} \right)}}{2m + 1}\frac{{\overline{\sigma }_{0} }}{E}\left( {\frac{3}{{\mu \left( {2m + 1} \right)}}\frac{{\overline{\sigma }_{0} }}{{\overline{\sigma }_{F} }}} \right)^{{\frac{\kappa }{m - 1}}} .$$

Figure 3 shows the analytical expression (17) together with the numerical results of the parametric study. Figure 3 illustrates that, despite the simplifying assumptions involved in the above derivation, the closed-form expression (17) is very accurate (the difference between analytical and numerical results is less than 10%) for highly squeezing ground conditions, where an extended region in front of the tunnel face exhibits elastoplastic deformations (*ρ*/*a* ≥ 1.5), and it is conservative in the case of moderate squeezing, where plastic yielding is limited to the vicinity of the face (1.5 ≥ *ρ*/*a* ≥ 1).

## Application example

The Gotthard base tunnel will be used as an example. The tunnel was excavated using TBMs, with the exception of the Sedrun section which was constructed by conventional tunnelling due to the expected heavy squeezing^22^. Considering the technical improvements of the last two decades^2^, a re-evaluation of the option of a TBM drive from today’s perspective is interesting. Here, we limit ourselves to the question of the extrusion-induced additional excavation volume; concerning the risk of shield jamming we refer to^23^.

**Fig. 7:**
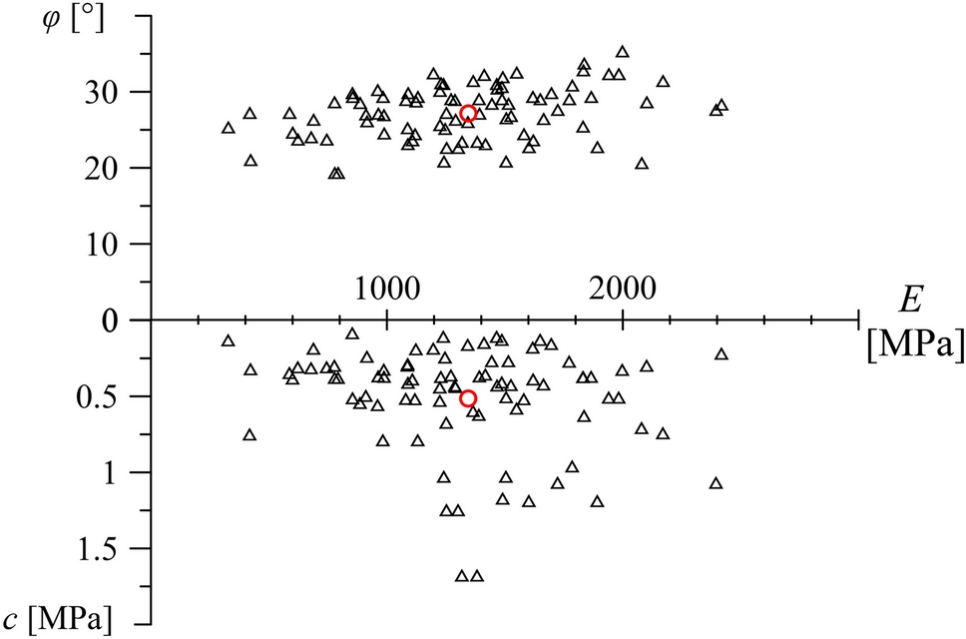
Friction angle *φ* and cohesion *c* over Young’s modulus *E* (86 tests after^16,19^).

In the Sedrun section the tunnel crosses kakirites, that are tectonically intensively sheared, weak and highly deformable rocks, at a depth of 800–900 m^24^. Information about the mechanical behaviour and parameters of the kakirites can be found in^16,19^. According to the results of consolidated-drained triaxial tests, the kakirites exhibit friction angles of 20°–30°, cohesions of 200–700 kPa and Young’s moduli of 800–1600 MPa (Fig. 7). The Young’s modulus of the kakirites depends on the confining pressure, obeying a power law with an exponent 0.78^19^; the *E*-values of Fig. 7 hold for the confining pressure at the depth of the tunnel (20 MPa).

**Fig. 8:**
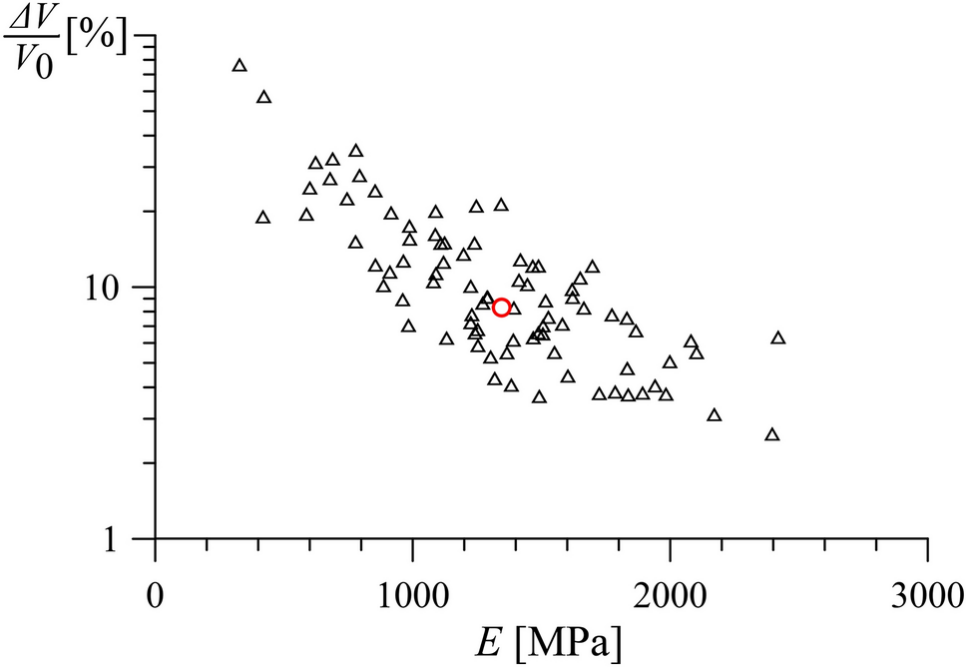
Normalized extrusion-induced excavation volume *ΔV*/*V*_0_ over Young’s modulus *E* for the 86 parameter sets of Fig. 7 (other parameters: *ψ* = 2°–11° after^19^, taken equal to 5° in the cases where it was not determined; *ν* = 0.25; *σ*_0_ = 20 MPa; *σ*_*F*_ = 0 MPa).

The additional excavation volume caused by the extrusion of the rock at the face is determined with Eq. (17) and presented in Fig. 8 as a function of the *E*-value of each parameter set. It mostly falls within the range 4–20% (average value of 9%). This result shows that the expected over-excavation is not critical for the conditions encountered in the Sedrun section of the Gotthard base tunnel, even when open mode operation is considered.

However, the additional excavation volume during advance may erroneously indicate unstable face conditions. The latter could be detected by reducing the excavation rate (by lowering the penetration rate) or even stopping the advance and monitoring the muck weight over time. A constant or increasing muck weight indicates instability.

In addition, although not necessary in the specific case, it can be quickly verified using Eq. (17) that closed-mode operation with face support could significantly reduce the over-excavation in the case of more severe squeezing. Therefore, the possibility of changing the mode of operation must be considered when selecting the TBM for tunnelling through heavily squeezing sections.

## Comparison with purely cohesive ground

The over-excavation *ΔV/V*_0_ after Eq. (17) is compared with that obtained for a purely cohesive ground of the same uniaxial compressive strength. According to^5^, the over-excavation *ΔV/V*_0_ can be expressed as

18 $$\frac{\Delta V}{{V_{0} }} = 0.35\left( {1 + \nu } \right)\frac{{f_{c} }}{E}e^{{\frac{{\sigma_{0} - \sigma_{F} }}{{f_{c} }}}}$$

for purely cohesive grounds obeying the Tresca yield criterion, considering *f*_*c*_ = 2*s*_*u*_ and *E* = *E*_*u*_(1 + *ν*)/1.5, where *s*_*u*_ and *E*_*u*_, denote the undrained shear strength and undrained Young’s modulus, respectively.

**Fig. 9:**
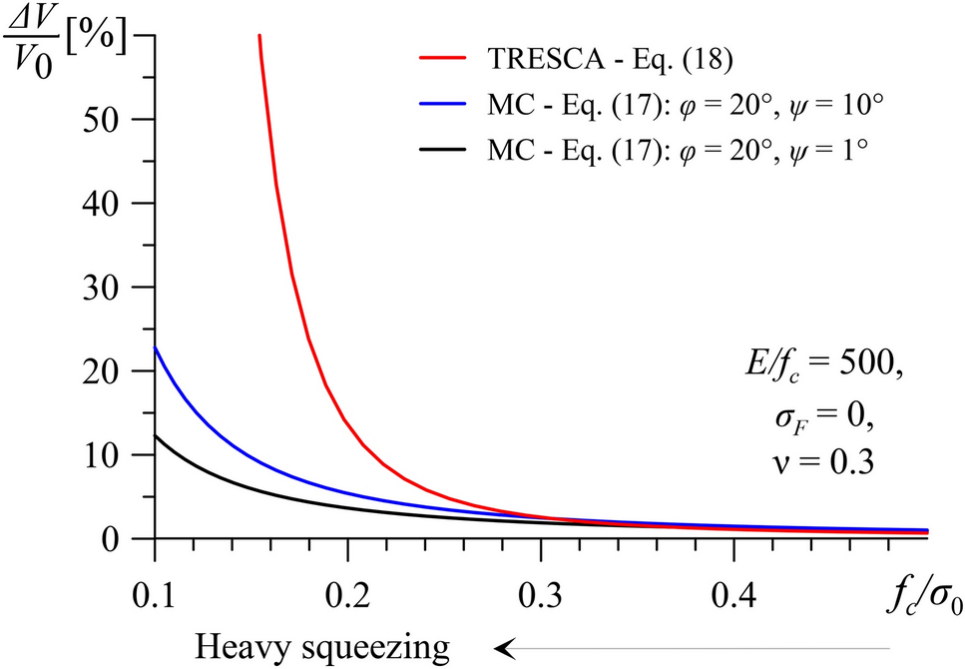
Normalized extrusion-induced excavation volume *ΔV*/*V*_*0*_ over normalized strength to in-situ stress *f*_*c*_/*σ*_*0*_ considering a purely-cohesive ground and a cohesive-frictional ground (other parameters: *E/f*_*c*_ = 500; *σ*_*F*_ = 0 MPa; ν = 0.3).

Figure 9 shows the over-excavation *ΔV/V*_0_ as a function of the normalised uniaxial compressive strength *f*_*c*_/*σ*_0_, assuming a constant stiffness to strength ratio *E*/*f*_*c*_ = 500, an unsupported face *σ*_*F*_ = 0 and a typical Poisson’s ratio of *ν* = 0.3. It is evident that the cohesive-frictional material is less susceptible to large over-excavation than the purely cohesive ground, especially if the former does not exhibit large dilatancy (i.e. *ψ* = 1°).

**Fig. 10:**
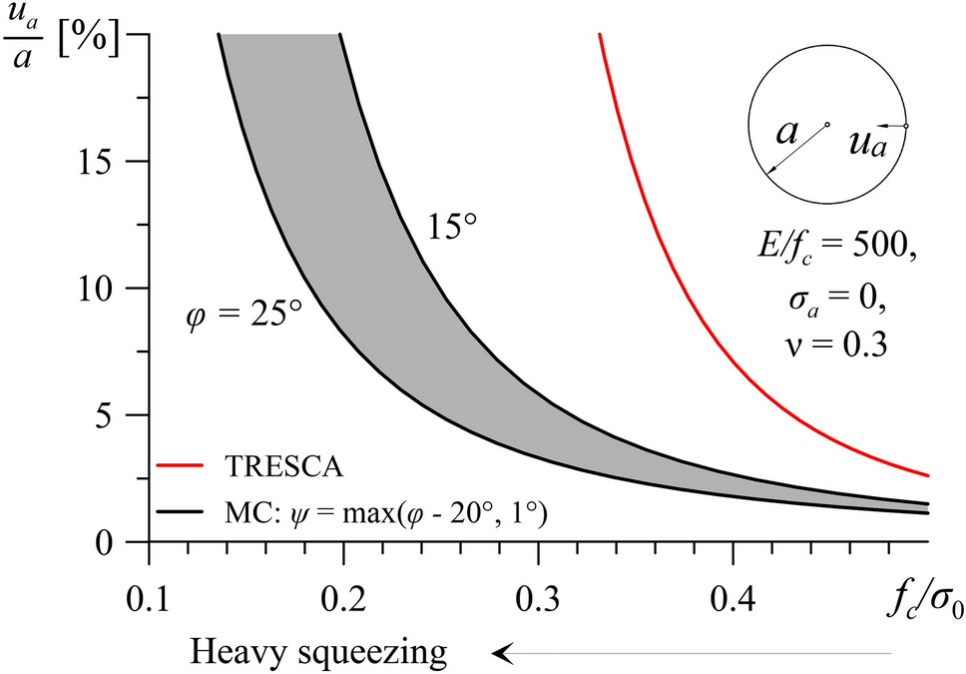
Normalized radial displacement *u*_*a*_/*a* of an unsupported cylindrical tunnel over normalized strength *f*_*c*_/*σ*_*0*_ (*E/f*_*c*_ = 500, ν = 0.3).

The results of Fig. 9 show similarities with those of the classical cavity contraction problem of an unsupported plane strain tunnel^25^. Figure 10 shows the normalised displacement of the tunnel wall *u*_*a*_/*a* as a function of the normalised uniaxial compressive strength *f*_*c*_/*σ*_0_, for a range of low friction angles (i.e. *φ* = 15°–25°) and Poisson’s ratio equal to *ν* = 0.3. In this case, too, the behaviour of purely cohesive ground is by far more unfavourable than that of a cohesive-frictional ground of the same normalised strength *f*_*c*_/*σ*_0_.

## Conclusions

This paper investigated the extrusion-induced additional excavation volume in mechanised tunnelling through squeezing ground obeying the Mohr–Coulomb yield criterion and quantifies for the first time the over-excavation at the face in cohesive-frictional grounds. Firstly, a comprehensive parametric study was performed using a computational method that accounts for the continuous removal of the extruding ground during the excavation process. The numerical results were presented in a single dimensionless chart. Secondly, a closed-form solution was derived for the excavation volume which, despite the underlying simplifications, accurately estimates the extrusion-induced excavation volume in highly squeezing ground. The usefulness of the developed equation was illustrated by a practical application example, the Sedrun section of the Gotthard Base Tunnel. Finally, it was shown that the behaviour of a purely cohesive ground is consistently more unfavourable than that of cohesive-frictional ground. This study shows quantitatively that over-excavation can be significantly reduced by supporting the tunnel face. This finding suggests that multi-mode TBMs may be the preferred choice for tunnelling through heavily squeezing grounds. From a practical point of view, a large over-excavation during advance can lead to (a) spoil logistics disruption, (b) cutterhead jamming and (c) misinterpretation of the spoil weight monitoring, each of which results in poor TBM performance in terms of rate of tunnel advance.

## Data Availability

Some or all data, models, or code that support the findings of this study are available from the corresponding author upon reasonable request.

## List of symbols

*a*: Tunnel radius
*E*: Young’s modulus
*E*_*u*_: Undrained Young’s modulus
*f*: A function
*f*_*c*_: Uniaxial compressive strength
*m*: Function of friction angle
*r*: Radial co-ordinate
*s*_*u*_: Undrained shear strength
*u*: Displacement
*u*_*C*_: Core displacement
*u*_*F*_: Face displacement
*u*_*x*_: Axial displacement
*v*: Tunnel advance rate
*V*_0_: Tunnel volume
*x*: Axial co-ordinate

## Greek symbols

*Δ**V*: Excess volume (“over-excavation”)
*Δ**x*: Numerical excavation step length
*ε*_*r*_: Radial strain
*ε*_*t*_: Tangential strain
*θ*: Polar co-ordinate
*κ*: Function of dilation angle
*μ*: Dimensionless stress ratio
*ν*: Poisson’s ratio
*ρ*: Radius of the elastoplastic zone
*σ*: Normal stress
$\overline{\sigma }$: Transformed normal stress
*σ*_*F*_: Face support pressure
*σ*_*r*_: Radial stress
*σ*_*s*_: Radial stress at *r* = *a*
*σ*_*ρ*_: Radial stress at the elastoplastic interface
*σ*_0_: Primary stress at tunnel axis
*τ*: Shear stress
*τ*_*π*__*/2*_: Shear stress at the edge of the face
*φ*: Friction angle
*ψ*: Dilation angle

## References

[1] Kovári, K. Tunnelling in squeezing rock. *Tunnel* **5**, 12–31 (1998).

[2] AFTES. État de l’art concernant les évolutions des tunneliers et de leurs capacités de 2000 à 2019. (2021).

[3] Anagnostou, G., Mezger, F., Pferdekämper, Th., Syomik, A. & Vrakas, A. *The Gibraltar Strait Tunnel - On the feasibility of mechanized tunnelling through the breccias*. ETH Zurich. Unpublished report prepared on behalf of SECEG SA, Madrid, Spain, and SNED, Rabat, Morocco (2018).

[4] Pferdekämper, T., Vrakas, A. & Anagnostou, G. Numerical modelling of face extrusion in shield tunnelling through squeezing ground. In *Challenges and Innovations in Geomechanics. IACMAG 2022. Lecture Notes in Civil Engineering* (eds Barla, M. et al.) (Springer, 2023).

[5] Pferdekämper, T., Vrakas, A. & Anagnostou, G. Interaction between TBMs and heavily squeezing purely cohesive grounds. *Géotechnique*10.1680/jgeot.24.01012 (2025).

[6] Pferdekämper, T. & Anagnostou, G. On the interplay between face extrusion and shield loading in squeezing conditions. In *Tunnelling for a better life* (eds Yan, J., Celestino, T., Thewes, M., Eberhardt, E.) Proceeding of the ITA-AITES World Tunnel Congress (WTC 2024), Shenzhen, China, 2056–2061 (2024).

[7] Zoppis, E. & Baldi, A. M. Gilgel Gibe II hydropower project in Ethiopia; TBM Tunnelling, when the rock turns into mud: Analysis of a major collapse, its causes and solutions. In *Geotechnical Aspects of Underground Construction in Soft Ground – Proc. of the 10th Int. Symp. on Geotech. Asp. of Under. Con. in Soft Ground, IS-CAMBRIDGE* (eds Elshafie, V. Mair) 2022, 199–207 (2021)

[8] Anagnostou, G. & Pferdekämper, T. Is face stability a problem in mechanised tunnelling through squeezing ground? In: *Tunnelling Into a Sustainable Future* Proceeding of the ITA-AITES World Tunnel Congress (WTC 2025), Stockholm, Sweden (accepted for publication, 2025).

[9] Dassault Systèmes. ABAQUS Analysis User’s Manual (2016).

[10] Anagnostou, G. & Kovári, K. Significant parameters in elastoplastic analysis of underground openings. *J. Geotech. Geoenviron. Engng.* **119**(3), 401–419 (1993).

[11] Vrakas, A. & Anagnostou, G. A finite strain closed-form solution for the elastoplastic ground response curve in tunnelling. *Int. J. Numer. Anal. Meth. Geomech.* **38**(11), 1131–1148 (2014).

[12] Anthi, M., Pferdekämper, T. & Anagnostou, G. Analytical solutions for cavity contraction in strain-softening materials with linear or exponential strength decay. *Sci. Rep.* **14**, 15040 (2024). 38951535 10.1038/s41598-024-65186-y PMC11217471

[13] Caquot, A. I. *Équilibre des massifs á frottement interne. Stabilité des terres pulvérulentes et cohérentes* (Gauthier-Villars, 1934).

[14] Anagnostou, G., Schuerch, R. & Perazzelli, P. Lake mead intake no 3 tunnel—design considerations and construction experiences. *Geomech. Tunnel.* **11**, 15–23 (2018).

[15] Vermeer, P. A. & de Borst, R. Non-associated plasticity for soils, concrete and rock. *Heron* **29**(3), 1–64 (1984).

[16] Vogelhuber, M., Pimentel, E. & Anagnostou, G. The strength, deformability and permeability of the kakiritic rocks from the Gotthard Base Tunnel. *J. Rock Mech. Geotech. Eng.* **15**(9), 2224–2237 (2023).

[17] Kovári, K. The determination of the characteristic line from straight line nomograms. In *Numerical methods in geomechanics* (eds Kawamoto & Ichikawa) **4**, 1741–1746 (1985).

[18] Egger, P. Deformation at the face of the heading and determination of the cohesion of the rock mass. *Undergr. Space.* **4**, 313–318 (1980).

[19] Vrakas, A., Dong, W. & Anagnostou, G. Elastic deformation modulus for estimating convergence when tunnelling through squeezing ground. *Géotechnique* **68**(8), 713–728 (2018).

[20] Anagnostou, G. Continuous tunnel excavation in a poro-elastoplastic medium. In *Numerical Models in Geomechanics - NUMOG X* (ed Pande & Pietruszczak), 183–188. Taylor & Francis Group, London (2007).

[21] Timoshenko, S. & Goodier, J. N. *Theory of Elasticity* (McGraw-Hill Book Company Inc, 1951).

[22] Kovári, K., Amberg, F. & Ehrbar, H. Mastering of squeezing rock in the Gotthard base. *World Tunnel.* **13**(5), 234–238 (2000).

[23] Leone, T., Nordas, A. & Anagnostou, G. Effects of creep on shield tunnelling through squeezing ground. *Rock Mech. Rock Eng.* **57**(1), 351–374 (2024). 38188540 10.1007/s00603-023-03505-x PMC10766731

[24] Mezger, F., Anagnostou, G. & Ziegler, H. The excavation-induced convergences in the Sedrun section of the Gotthard Base Tunnel. *Tunnel. Undergr. Space Technol.* **38**, 447–463 (2013).

[25] Salençon, J. Contraction quasistatique d’une cavité à symétrie sphérique ou cylindrique dans un milieu elastoplastique. *Annales des Ponts et Chaussées* **4**, 213–236 (1969).

